# Multiplexed Promoter Engineering for Improving Thaxtomin A Production in Heterologous *Streptomyces* Hosts

**DOI:** 10.3390/life12050689

**Published:** 2022-05-06

**Authors:** Xuejin Zhao, Yeqing Zong, Weijia Wei, Chunbo Lou

**Affiliations:** 1 CAS Key Laboratory of Microbial Physiological and Metabolic Engineering, Institute of Microbiology, Chinese Academy of Sciences, Beijing 100101, China; zhaoxj@im.ac.cn (X.Z.); zongyeqing@bluepha.com (Y.Z.); weiweijia15@mails.ucas.ac.cn (W.W.); 2 State Key Laboratory of Microbial Resources, Institute of Microbiology, Chinese Academy of Sciences, Beijing 100101, China; 3 College of Life Sciences, University of Chinese Academy of Sciences, Beijing 100149, China; 4 Center for Cell and Gene Circuit Design, CAS Key Laboratory of Quantitative Engineering Biology, Shenzhen Institute of Synthetic Biology, Shenzhen Institutes of Advanced Technology, Chinese Academy of Sciences, Shenzhen 518055, China; 5 Guangdong Provincial Key Laboratory of Synthetic Genomics, Shenzhen Institute of Synthetic Biology, Shenzhen Institutes of Advanced Technology, Chinese Academy of Sciences, Shenzhen 518055, China; 6 Shenzhen Key Laboratory of Synthetic Genomics, Shenzhen Institute of Synthetic Biology, Shenzhen Institutes of Advanced Technology, Chinese Academy of Sciences, Shenzhen 518055, China

**Keywords:** thaxtomin A, gene cluster, heterologous expression, multiplexed promoter engineering

## Abstract

Thaxtomin A is a potent bioherbicide in both organic and conventional agriculture; however, its low yield hinders its wide application. Here, we report the direct cloning and heterologous expression of the thaxtomin A gene cluster in three well-characterized *Streptomyces* hosts. Then, we present an efficient, markerless and multiplex large gene cluster editing method based on in vitro CRISPR/Cas9 digestion and yeast homologous recombination. With this method, we successfully engineered the thaxtomin A cluster by simultaneously replacing the native promoters of the *txtED* operon, *txtABH* operon and *txtC* gene with strong constitutive promoters, and the yield of thaxtomin A improved to 289.5 µg/mL in heterologous *Streptomyces coelicolor* M1154. To further optimize the biosynthetic pathway, we used constraint-based combinatorial design to build 27 refactored gene clusters by varying the promoter strength of every operon, and the highest titer of thaxtomin A production reached 504.6 μg/mL. Taken altogether, this work puts forward a multiplexed promoter engineering strategy to engineer secondary metabolism gene clusters for efficiently improving fermentation titers.

## 1. Introduction

A number of *Streptomyces* interact directly with plants or have indirect effects on plant health and growth through mechanisms such as biofertilization or biocontrol [1,2,3]. Specifically, Russell et al. discussed six different potato scab pathogenic *Streptomyces* strains from various niches that produce at least 11 thaxtomins, which are cyclic dipeptides (2,5-diketopiperazines) with a unique 4-nitroindole moiety [4]. Their structural diversity lies in the presence or absence of *N*-methyl and/or hydroxyl groups at different sites (Figure S1A). Among them, thaxtomin A is the major product that displays good cellulose synthesis inhibition activity. It could efficiently reduce the germination and growth of green algae, blue algae, and red algae [5] and deter weed germination [6]. The 20-kb thaxtomin A gene cluster encodes seven proteins responsible for the regulation and biosynthesis of thaxtomin A (Figure S1B,C). TxtD, a nitric oxide synthase, generates nitric oxide (NO) from L-arginine, which is then used by cytochromes P450 enzyme (TxtE) to nitrate C-4 of L-tryptophan, resulting in 4-NO_2_-L-tryptophan [7,8]. Two nonribosomal peptide synthetases, TxtA and TxtB, cyclize 4-NO_2_-L-tryptophan with L-phenylalanine to form a diketopiperazine thaxtomin D [9], which is further hydroxylated twice by another P450, TxtC, to produce thaxtomin A [10]. In addition, one MbtH-like protein (TxtH) assists the proper folding of TxtA and TxtB [11]. TxtR, an AraC/XylS family transcriptional activator, functions as the pathway-specific regulator for thaxtomin A production in response to cello-oligosaccharides [12]. Considering its attractive environmental compatibility and rapid degradation in the natural environment, in 2012, thaxtomin A was approved as a biodegradable bioherbicide by the U.S. Environmental Protection Agency [13]. However, the low yield in its native *Streptomyces* producer has greatly hindered its applications in agriculture. In 2018, Jiang et al. expressed the thaxtomin gene cluster in a heterologous host *Streptomyces albus* J1074 and described a high-yield production of thaxtomin A (168 μg/mL) after further medium optimization [14]. In a recent study, Li et al. employed a stepwise strategy by combining heterologous expression, repressor deletion and activator overexpression to increase the production of thaxtomin A to 161 μg/mL in shake-flask cultures. After optimization of the culture media, the maximum yield of thaxtomin A and its analogs reached 728 μg/mL in a 5 L stirred-tank bioreactor [15].

Synthetic biology approaches allow us to reengineer gene clusters in a way to disrupt native transcriptional regulation systems by synthetic regulation elements to awaken or improve target compound production [16,17]. Among them, promoter engineering has been widely used to modify the genetic regulation of a given cluster with a set of well-characterized promoters. For example, we activated and overproduced the cryptic lycopene in *Streptomyces avermitilis* by replacing the natural promoter with a strong synthetic promoter [18]. Moreover, most natural product BGCs are composed of multiple operons, and thus their activation requires the replacement of several promoters in a single gene cluster. For instance, the silent streptophenazine BGC from the marine actinomycete *Streptomyces* sp. CNB-091 was activated by introducing four constitutive promoters (*ermE^∗^p/actIp/sp44/p21*) at different positions in the BGC [19]. Similarly, the silent spectinabilin gene cluster was awakened in a heterologous host by the replacement of each of the native promoters with characterized strong promoters [20]. For rapid engineering of the large gene cluster, efficient multi-site gene cluster editing methods, such as mCRISTAR [21], *mi*CASTAR [22] and mpCRISTAR [23], were developed and employed to activate the silent tetarimycin A (Tam) gene cluster by inserting eight promoter cassettes. Compared to mCRISTAR and mpCRISTAR, which are based on in vivo gene cluster digestion in yeast, *mi*CASTAR based on in vitro digestion is simpler, more cost-effective and more time-saving. However, to achieve high-efficiency multiplexing, *mi*CASTAR requires at least one promoter cassette to combine autotrophic markers. This inevitably limits the scalability of multiplexed gene cluster engineering approaches.

Here, we report the capture, engineering and heterologous expression of the thaxtomin A gene cluster from *S.* *acidisabies* ATCC 49003 in three well-characterized *Streptomyces* hosts, and the yield of thaxtomin A was remarkably enhanced by refactoring the gene cluster with strong constitutive promoters using a markerless and multi-site editing method. Moreover, we further combinatorically optimized the production of thaxtomin A by constructing a pathway library to balance the expression of *txtED, txtABH* and *txtC* operons with promoters of different strengths. This work provides an efficient strategy for not only improving known bioactive natural products but also discovering new families of bioactive natural products.

## 2. Materials and Methods

### 2.1. Strains, Media and Chemicals

The strains used in this study are summarized in Table 1*. Escherichia coli* DH10B was used for molecular cloning and plasmid propagation throughout this study. *E. coli* ET12567/pUZ8002 [24] was used for *E. coli-Streptomyces* conjugation. LB medium supplemented with appropriate antibiotic (50 μg/mL apramycin) was used for *E. coli* growth. *Saccharomyces cerevisiae* VL6-48 [25] was used for thaxtomin A gene cluster editing and grown in YPD medium (2 g/L glucose, 1 g/L yeast extract, 2 g/L peptone and 0.8 g/L adenine sulfate). Yeast transformants were selected on synthetic dropout media (1.7 g/L yeast nitrogenous base, 5 g/L ammonium sulfate, 20 g/L yeast SD media supplements (Sigma, without uracil), 20 g/L dextrose, and 20 g/L agar).

*S.acidisabies* and *S.* *albus* strains were grown in Mannitol Soya Flour (MS) medium (20 g/L soybean flour, 20 g/L mannitol and 20 g/L agar) for spore preparation. *S.* *venezuelae* strains were cultivated on Maltose-Yeast Extract-Malt Extract (MYM) medium (4.2 g/L (D-(+)-maltose monohydrate, 4 g/L yeast extract, 4 g/L malt extract and 20 g/L agar) for spore preparation. For seed cultivation, *S. venezuelae* strains were grown in liquid MYM, while *S.* *acidisabies*, *S. coelicolor* and *S.* *albus* strains were grown in Trypticase Soy Agar (TSB) (30 g/L tryptic soy broth (BD)). Medium Oat Bran Broth OBB (20 g/L Oat, pH 7.2) was made for thaxtomin A production.

### 2.2. Cloning the Thaxtomin A Gene Cluster

The primers used in this study are summarized in Table S1. The thaxtomin A gene cluster from *S. acidiscabies* ATCC 49003 was directly cloned according to a previously described method [29]. Mycelia of *S. acidiscabies* ATCC 49003 were collected after 2 days of cultivation in TSB media at 30 °C. Preparation of the genomic DNA plugs was carried out following the instruction manual of the CHEF genomic DNA plug kit (Bio–Rad, Hercules, CA, USA). The *S. pyogenes* Cas9 protein expression plasmid was provided by Professor Zhen Xie (Tsinghua University), and the His-tagged Cas9 protein was first purified by Ni-NTA affinity chromatography and then further purified by Mono S column [18]. The DNA templates of sgRNA-thax-F and sgRNA-thax-R were generated via overlap extension of sgRNA-thax-up/sgRNA-thax-down and guide RNA-F plus guide RNA-R, respectively (Table S1). PCR reactions contained 25 µL 2× Taq PCR MasterMix (Biomed, London, UK), 1 µL of each primer (10 µM stocks) and 22 µL water. The PCR program was as follows: 95 °C for 3 min, followed by 20 cycles of 95 °C for 30 s, 55 °C for 30 s and 72 °C for 15 s, followed by a 5-min extension at 72 °C. Then, the PCR products were purified using a DNA purification kit (Biomed). In vitro transcription of sgRNAs was performed with the HiScribe™ T7 Quick High Yield RNA Synthesis Kit (NEB). sgRNAs were purified using the RNApure Rapid RNA Kit (BioMed). Then, the DNA plugs were digested with 500 ng Cas9 guided by 500 ng sgRNA-F and 500 ng sgRNA-R at 37 °C for 2 h, and the digested DNA was precipitated with ethanol and resuspended in 20 µL DNase-free water. The linearized capture vector pPAS was amplified using the primers thax-vec-F and thax-vec-R (Table S1) to introduce two ~30 bp overlaps with the corresponding ends of the thaxtomin gene cluster fragment. Approximately 50 ng of the pPAS backbone and 1 µg of digested genome fragments were assembled using Gibson Assembly and introduced into *E. coli* DH10B by electroporation. The correct recombinant plasmid was verified by PCR using the PF1 and PR1, PF2 and PR2, PF3 and PR3, and PF4 and PR4 primers (Table S1). PCR reactions contained 12.5 µL 2× Taq PCR MasterMix (Biomed), 0.5 µL of each primer (10 µM stocks), 1 μL plasmid (1 ng/μL) up to 25 µL final volume with water. The PCR program was as follows: 95 ℃ for 3 min, followed by 32 cycles of 95 °C for 30 s, 60 °C for 30 s and 72 °C for 1 min, followed by a 5-min extension at 72 °C. PCR products were detected by agarose gel electrophoresis and DNA sequencing. Then, the recombinant plasmid was further confirmed by restriction digestion using NotI, NheI and AflII (NEB, Ipswich, MA, USA).

### 2.3. Refactoring of the Thaxtomin A Gene Cluster with Constitutive Promoters

Promoters picked from the *kasO*p*-based synthetic promoter library [18] were used to overexpress the thaxtomin A gene cluster (Table S2). Three CRISPR target sequences were selected from three promoter regions (s1 for *txtED*, s2 for *txtABH*, s3 for *txtC*) in the thaxtomin A cluster, and one target sequence (s4) for inserting an auxotrophic marker was selected from vector pPAS. The DNA templates of sgRNA-s1 and sgRNA-s2, and sgRNA-s3 and sgRNA-s4 were generated via overlap extension of sgRNA-s(x)f and guide RNA-F plus guide RNA-R, respectively. In vitro transcription of sgRNAs was performed with the HiScribe™ T7 Quick High Yield RNA Synthesis Kit. For the promoter refactoring of *txtED* and *txtABH*, the pPAS-thax plasmid (10 µg) was digested with 1 µg Cas9 guided by three sgRNAs (including 1 µg sgRNA-s4, 1 µg sgRNA-s1 and 1 µg sgRNA-s2) at 37 °C for 2 h. To simultaneously refactor the promoters of *txtED*, *txtABH* and *txtC*, four sgRNAs (including sgRNA-s4, sgRNA-s1, sgRNA-s2 and sgRNA-s3) were employed. After digestion, the DNA was precipitated with ethanol, and 1 µg was used in the following steps. The yeast autotrophic marker (URA) was amplified from plasmid pESC-URA (GenScript, Piscataway, NJ, USA) using primers containing 40 bp sequences matching the targeted insertion site s4. The promoters were amplified by PCR using the specific primers listed in Table S1. Approximately 150–300 ng of the purified PCR products and 1 µg of the digested pPAS-thax fragments were cotransformed into *S. cerevisiae* VL6-48 using the Frozen-EZ Yeast Transformation II Kit (Zymo Research, Irvine, CA, USA). The correct promoter insertion was screened by PCR using primers up- and downstream of the target site txtEA-F and txtEA-F, and txtC-F and txtC-R (Table S1). Correct plasmids were isolated and transferred into ET12567/pUZ8002 and then moved to *S. coelicolor* M1154, *S. albus* J1074 and *S. venezuelae* ISP5230 by intergeneric conjugation, and the resulting strains are listed in Table 1.

### 2.4. Thaxtomin A Production and HPLC Analysis

*S. albus* and *S. coelicolor* strains were cultured in TSB medium at 30 °C for 48 h, and *S. venezuelae* strains were cultured in MYM medium at 30 °C for 48 h. Then, 2 mL of the resultant seed cultures was used to inoculate 50 mL of OBB medium in 250 mL flasks. The cultures were incubated at 30 °C and 250 rpm for 5 days. For thaxtomin A detection, 1 mL of fermentation broth was extracted with 10 mL of methanol, and the mycelium was removed by centrifugation at 8000 rpm for 10 min, and the supernatants were filtered through a 0.22 μm pore-size nylon membrane. High-performance liquid chromatography (HPLC) analysis was performed with an Agilent 1100 HPLC system (C18 column: 5 μm × 250 mm × 4.6 mm; mobile phase: acetonitrile: water (40:60), 1 mL/min; detected at 380 nm). Thaxtomin A standard was purchased from Sigma (catalog: SML1456).

## 3. Results

### 3.1. Direct Cloning and Heterologous Expression of the Thaxtomin A Gene Cluster

**Figure 1:**
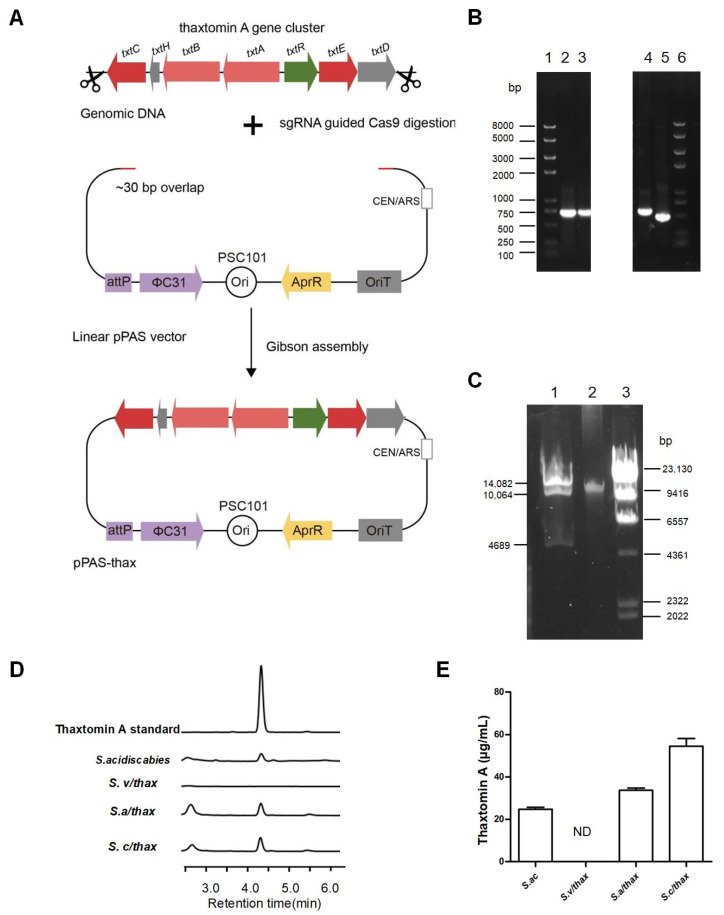
Cloning and heterologous expression of the thaxtomin A gene cluster. (**A**) Schematic representation of the strategy for direct cloning of the thaxtomin A gene cluster using the CATCH method. The genomic DNA was cut by sgRNA-guided Cas9 and then inserted into the linearized pPAS vector via Gibson assembly. (**B**) Confirmation of the plasmid pPAS-thax by PCR. The 5′ region was PCR by the primer pair PF1and PR1. PCR verification of the 3′ region was performed by the primer pair PF2and PR2. PCR verification of gene cluster internal genes was performed by the primer pairs PF3and PR3, and PF4and PR4. M: Trans2K^®^ Plus DNA Marker; 1: 5′ region PCR product (702 bp); 2: 3′ region PCR product (710 bp); 3: *txtE* internal region PCR product (530 bp); 4: *txtB* internal region PCR product (449 bp). (**C**) Confirmation of the plasmid pPAS-thax by restriction enzyme digestion. M: λ DNA/HindIII marker; 1: pPAS-thax plasmid; 2: pPAS-thax plasmid digested by NheI + AflII+ NotI. (**D**) HPLC analysis of thaxtomin A production in *S.* *acidsabies* ATCC 49003 and heterologous expression in *S.* *coelicolor* M1154, *S.* *albus* J1074 and *S.* *venezuelae* ISP5230. (**E**) Comparison of thaxtomin A yields in three *Streptomyces* heterologous hosts. ND, not detected. The data represent three biological replicates.

**Figure 2:**
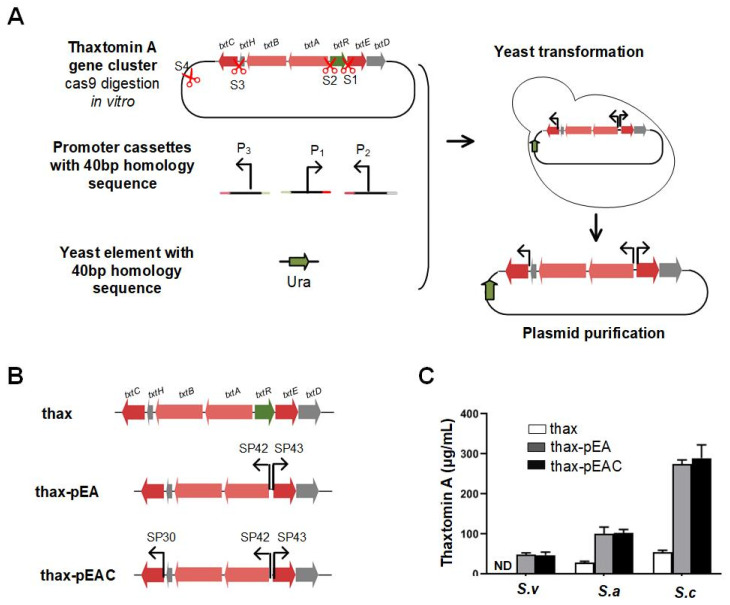
Refractory thaxtomin A gene cluster using strong constitutive promoters. (**A**) Schematic illustration of the replacement of native promoters of the thaxtomin A gene cluster with strong *kasO*p*-based synthetic promoters [17]. (**B**) In the thax-pEA construct, the promoters of *txtDE* and *txtABH* were replaced with SP43 andSP42, respectively. In the thax-pEAC construct, the promoters of *txtDE*, *txtABH* and *txt*C were replaced with SP43, SP42 and SP30, respectively. (**C**) Yield analysis of the two refactored gene clusters with strong constitutive promoters in different *Streptomyces* heterologous hosts. ND, not detected. Data represent three biological replicates.

Vector pPAS was first constructed for gene cluster cloning and editing, and contains a yeast origin of replication (CEN/ARS), a low copy origin of replication (pSC101) for *E.* *coli*, an origin of transfer (oriT) for *E.* *coli-Streptomyces* conjugation, the *Streptomyces* ΦC31 attP-int system and the *Streptomyces* apramycin resistance gene. We cloned the thaxtomin A gene cluster from *S. acidiscabies* ATCC 49003 into the pPAS vector by the Cas9-assisted targeting of chromosome segments approach (CATCH) developed in our previous work [29] (Figure 1A). The resultant plasmid pPAS-thax containing the whole thaxtomin A gene cluster was verified by PCR amplification and restriction digestions (Figure 1B,C). To search for a suitable heterologous host for thaxtomin A production, pPAS-thax and the control plasmid pPAS were introduced into three model *Streptomyces* hosts—*S. venezuelae* ISP5230 [26], *S. albus* J1074 [27] and *S. coelicolor* M1154 [28]—by *E. coli-Streptomyces* conjugation. The resulting recombinant strains *S.v*/thax, *S.a*/thax, *S.c*/thax, *S.v/*pPAS, *S.a*/pPAS and *S.c*/pPAS and the original strain *S. acidiscabies* ATCC 49003 were fermented using OBB medium. After five days of fermentation, the culture extracts were analyzed by high-performance liquid chromatography (HPLC) (Figure 1D). As displayed in Figure 2E, in comparison with the native host *S. acidscabies* (23.23 μg/mL), the highest production of thaxtomin A was detected in *S. coelicolor* M1154 (55.07 μg/mL), followed by *S. albus* J1074 (34.87 μg/mL). However, no thaxtomin A was detected in *S.v*/thax or any control strains. These results demonstrated that the thaxtomin A gene cluster was expressed successfully in two heterologous hosts, especially in *S. coelicolor* M1154.

### 3.2. Enhanced Thaxtomin A Production by Refactoring the Thaxtomin A Gene Cluster Using Strong Constitutive Promoters

**Figure 3:**
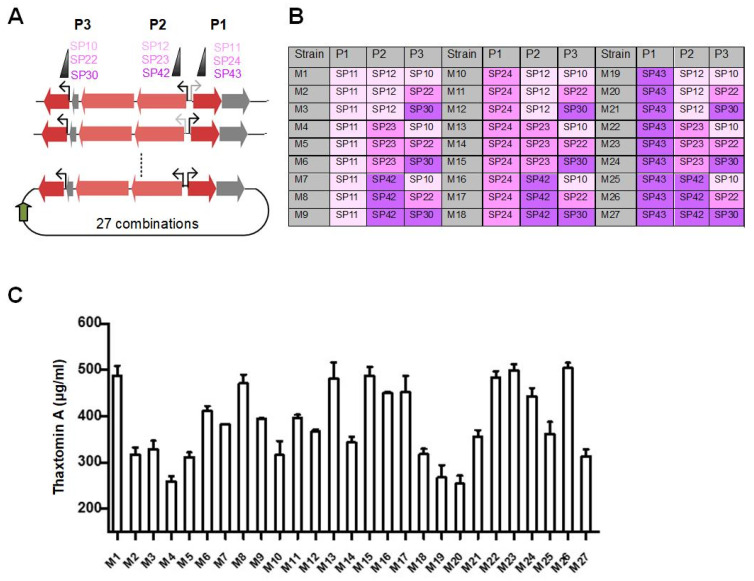
Combinatorial promoter engineering of the thaxtomin A gene cluster. (**A**) Overview of our combinatorial promoter engineering strategy. Promoters with different strengths were used to generate the library of refactored constructs. All promoter sequences are shown in Table S2. (**B**) Summary of *S. coelicolor* M1154 strains harboring the refactored thaxtomin A gene clusters. 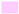: weak promoter; 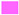: medium strength promoter; 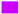: strong promoter. (**C**) The yields of thaxtomin A of the 27 resultant variants of *S. coelicolor*. Data represent three biological replicates.

The thaxtomin A gene cluster is predicted to contain three biosynthetic operons driven by three promoters and regulated by the pathway-specific transcriptional regulator TxtR. To reduce the complexity of the regulation hierarchy, we planned to delete the *txtR* gene and introduce strong constitutive promoter cassettes upstream of *txtABH, txtED* and *txtC*. To accomplish this more efficiently, a multiplexed gene cluster editing strategy that combined CRISPR/Cas9 with TAR was developed. The workflow of this method is shown in Figure 2A. In the first step, the plasmid carrying gene cluster was digested in vitro using sgRNA-directed Cas9 enzyme, and the digested fragments were recovered by precipitation using ethanol. Promoter cassettes and auxotrophic marker containing 40 bp homologous sequences matching each Cas9 digestion site were amplified by PCR. To limit the possibility of creating false-positive colonies and achieve markerless gene cluster editing, the auxotrophic marker did not combine with any of the insertion promoter fragments or yeast replication elements, and was designed to insert into the independent s1 site of the pPAS vector. In the second step, promoter cassettes, auxotrophic markers and the digested plasmid fragments were cotransformed into *S. cerevisiae* using a yeast transformation kit, and the transformants were selected on drop-out SC agar plates and verified by PCR. For promoter engineering of the thaxtomin A gene cluster, we first introduced two strong *KasO*p*-based promoters, SP43 and SP42, upstream of *txtED* and *txtABH*, and deleted the *txtR* regulator gene simultaneously (Figure 2B). For this study, pPAS-thax was digested with Cas9 guided by three sgRNAs (s1, s2 and s4); then, the digested fragments, SP43 and SP42 promoters, and URA auxotrophic marker were cotransformed into yeast. The resulting colonies were picked and analyzed by PCR, and the recombineering efficiency achieved 100% for simultaneous insertion of the SP42 and SP43 promoters and deletion of the *txtR* gene to generate the refactored gene cluster thax-pEA (Figure 2B). Then, to completely refactor the thaxtomin A gene cluster, the SP43 and SP42 promoter cassettes and an additional strong constitutive promoter cassette SP30 inserted upstream of *txtC* were cotransformed into yeast with the corresponding pPAS-thax fragments digested with Cas9 guided by four sgRNAs (s1, s2, s3 and s4). Efficiencies of 75% were achieved for three promoter cassette insertion experiments, and the refactored cluster was named thax-pEAC (Figure 3B). Next, the two recombinant plasmids thax-EA and thax-pEAC were moved from yeast through *E.* *coli* into *S. albus* J1074, *S. coelicolor* M1154 and *S. venezulea* ISP5230 for heterologous expression. Compared with the wild-type gene cluster, the refactored gene cluster thax-EA resulted in approximately 2.6-fold and 4-fold enhancement of thaxtomin A production in *S. albus* J1074 and *S. coelicolor* M1154, respectively (Figure 2C). Meanwhile, thaxtomin A production was successfully activated in *S.* *venezuelae* ISP5230, and the yield climbed to 49 μg/mL (Figure 2C). In addition, thax-pEAC resulted in a higher yield (289.5 μg/mL) in *S. coelicolor* M1154, whereas the amounts of thaxtomin A were comparable to thax-pEA and thax-pEAC in *S. albus* J1074 and *S. venezuelae* ISP5230 (Figure 2C). The results clearly indicate that overexpression of *txtABH* and *txtED* operons with a strong constitutive promoter significantly increased the production of thaxtomin A. Moreover, in the *S. coelicolor* M1154 host, an additional strong constitutive promoter to overexpress *txtC* can further slightly enhance thaxtomin A synthesis.

### 3.3. Refactoring of Thaxtomin A Gene Cluster Using Promoter Library

Although overexpression of biosynthesis genes might efficiently increase productivity in many cases, the balanced expression of multiple genes is central for pathway optimization and important to maximize end-product production [30]. Here, this fine-tuning was obtained through a combinatorial design based on a synthetic promoter library with different transcriptional strengths. Nine promoters were selected from the synthetic promoter library [18]: SP43, SP42 and SP30, corresponding to high expression; SP24, SP23 and SP22, corresponding to medium expression; and SP11, SP12 and SP10, corresponding to low expression (Figure 3A) (Table S2). Based on our combinatorial design, 27 different constructs pPAS-thax-M(x) with diverse combinations of promoters were generated (Figure 3B). After transformation to *S. coelicolor* M1154, a further increase in thaxtomin A was observed from 20 constructs, and the highest thaxtomin A titer achieved 504.6 μg/mL in strain M26, which harbors the strong promoter SP43 on *txtED*, strong promoter SP42 on *txtABC* and medium-strength promoter SP22 on *txtC* (Figure 3C). We analyzed the library to see whether there were correlations between thaxtomin A yield and promoter combinations. We found that *txtA* and *txtE* required stronger promoters to significantly promote the biosynthesis of thaxtomin A. In contrast, low or medium strength is preferable to *txtC*, indicating that the cellular availability of txtC may not limit the productivity of thaxtomin A and, more likely, it is in the same operon with *txtAB*. In addition, we found that weak promoters for *txtE* always paired with strong expression of *txtC* among all the higher thaxtomin A production combinations, and vice versa. There might be a delicate regulation and balance between TxtE and TxtC. However, it is noteworthy that the M1 had high thaxtomin A yield, but with weak promoters for all operons.

## 4. Discussion

Thaxtomin A possesses significant agricultural applications for weed control. Although efforts have been invested in improving its yield, there is still a large amount of work needed to meet the ever-increasing demand. To this end, we tried to express the thaxtomin A gene cluster in three different *Streptomyces* hosts. Our results indicate that the production of thaxtomin A was higher in both *S. coelicolor* M1154 and *S. albus* J1074 than in the native producer, but the cluster was silent in *S. venezuelae* ISP5230. In accordance with the complicated regulation of secondary metabolism in *Streptomyces* [31], thaxtomins production is indeed under strict regulation at multiple levels [12,32]. In different heterologous hosts, TxtR might be controlled directly or indirectly by some different trans-acting regulators and lead to various expression levels of thaxtomin A. Because understanding the regulation hierarchy remains a challenging issue, synthetic biology provides rational engineering principles for refactoring BGCs with well-characterized promoters to induce or improve secondary metabolite production [33]. Promoter engineering strategy allows for the overproduction of valuable metabolites [34]. However, BGC refactoring for multiplexed promoter engineering is always time-consuming and labor-intensive, and relies on the incorporation of selectable markers [21,22,23]. Here, we provide an easy and efficient approach to accomplish multiple promoter engineering. With comparable efficiencies, our strategy is completely markerless. We applied this strategy for combinatorial optimization of the thaxtomin A gene cluster and markedly enhanced the heterologous production of thaxtomin A, resulting in up to a 20-fold improvement in the thaxtomin A titer compared to the original strain. Compared to the traditional gene engineering in most wild-type producers, our strategy would effectively and easily optimize the production of secondary metabolites through precise regulation of BGCs by promoter engineering. Moreover, our results demonstrated that balancing the enzyme levels by combinatorial promoter engineering could significantly increase the production of end-up secondary metabolites. However, very few general rules for the assembly of thaxtomin A gene cluster were gleaned. Further work such as RNA-Seq is needed to determine the correlation between the transcriptional levels of every gene and thaxtomin A titer. Moreover, besides transcriptional regulation, it is important to regulate gene expression at the translation level [34]. Indeed, Horbal et al. reported that the enhancement of bottromycin production is not strictly proportional to the high transcriptional levels of genes. They speculated that the translation levels of genes also influence bottromycin production [35]. Indeed, another paper revealed that *btmC* transcription level was not correlated to bottromycin production, but strong relativity existed between *btmC* translation level and bottromycin biosynthesis. They used a theophylline inducible riboswitch to control the bottromycin pathway, resulting in a 120-fold bottromycin increase [36]. RBS library is also often applied for pathway optimization by varying the expression of enzymes at the translational level [37]. In order to decrease the interfere between promotes and RBSs, Bai et al. found that RiboJ insulator could improve the designability of gene cluster in *Streptomyces* [18]. In all, promoter engineering is an effective strategy for the optimization of gene clusters to improve the production of valuable metabolites. However, other important factors such as translation level should also be considered to precisely control gene cluster expression.

## 5. Conclusions

In this study, we reported a strategy to activate and optimize the cryptic biosynthesis gene cluster based a simple, efficient, markerless and high-throughput editing approach. Then, we combinatorically optimized the production of thaxtomin A by constructing a pathway library to balance the expression of *txtED*, *txtABH* and *txtC* operons with fine-tuned promoters. The best combination for thaxtomin A was enhanced to 504.62 μg/mL in the heterologous host *Streptomyces coelicolor* M1154. Altogether, the approach we have demonstrated here could be used as a general strategy for the development of high-titer industrial strains of valuable secondary metabolites produced by multi-operon biosynthetic systems.

## Figures and Tables

**Table 1:** Strains and plasmids used in this study.

Strains/Plasmids	Relevant Genotype	References or Source
**Plasmids**		
pPAS	Shuttle vector, containing *E. coli* origin of replication, pSC101, apramycin resistance, φC31 integration system	This study
pPAS-thax	pPAS harboring thaxtomin A gene cluster	This study
pPAS-thax-pEA	A derivative of pPAS-thax with replacement of the promoters of *txtED* and *txtABH* by SP43 and SP42	This study
pPAS-thax-pEA	A derivative of pPAS-thax with replacement of the promoters of *txtED*, *txtABH* and *txtC* by SP43, SP42 and SP30	This study
pPAS-thax-M(x)	A derivative of pPAS-thax with replacement of the promoters of *txtED*, *txtABH* and *txtC* by different *kas*Op*-based synthetic promoters	This study
**Strains**		
*E. coli* DH10B	Host for molecular cloning	Thermo Scientific
*E. coli* ET12567/pUZ8002	Donor strain for conjugation between *E. coli* and *Streptomyces*	[24]
*S. cerevisiae* VL6-48	The homologous recombination host for DNA assembly, MAT*α*, *his3-*Δ*200*, *trp1-*Δ*1*, *ura3-52*, *lys2*, *ade2-101*, *met14*, *psi* + *cir^0^*	[25]
*S. acidiscabies* ATCC 49003	Native thaxtomin A producer	CGMCC 4.1789
*S. venezuelae* ISP5230	Wild type, heterologous host	[26]
*S. albus* J1074	Wild type, heterologous host	[27]
*S. coelicolor* M1154	Heterologous host, *S. coelicolor* derivative (∆*act* ∆*red* ∆*cpk* ∆*cda rpoB*(C1298T) *rpsL*(A262G))	[28]
*S.a*/thax-pEA	Heterologous host *S*. *albus* J1074 containing pPAS-thax-pEA	This study
*S.c*/thax-pEA	Heterologous host *S. coelicolor* M1154 containing pPAS-thax-pEA	This study
*S.v*/thax-pEAC	Heterologous host *S. venezuelae* ISP5230 containing pPAS-thax-pEAC	This study
*S.a*/thax-pEAC	Heterologous host *S*. *albus* J1074 containing pPAS-thax-pEAC	This study
*S.c*/thax-pEAC	Heterologous host *S. coelicolor* M1154 containing pPAS-thax-pEAC	This study
M1-M27	Heterologous host *S. coelicolor* M1154 containing refactored thaxtomin A gene cluster pPAS-thax-M(x)	This study

## Data Availability

Data that support the finding of this study are available in the main text and the Supplementary Materials.

## Supplementary Materials

The following supporting information can be downloaded at: https://www.mdpi.com/article/10.3390/life12050689/s1, Figure S1: Thaxtomins and the biosynthetic pathway of thaxtomin A; Table S1: PCR primers used in this study; Table S2: Sequence of promoters used in this study.
