# Supplementary material for: Multiplexed Promoter Engineering for Improving Thaxtomin A Production in Heterologous Streptomyces Hosts
Source: Life (Basel). 2022 May 6;12(5):689. doi: 10.3390/life12050689 (PMC9146380; doi:10.3390/life12050689)
Supplement: Supplementary file 1 [file life-12-00689-s001.zip › life-1671854-supplementary.pdf]

life

MDPI

Supporting Information

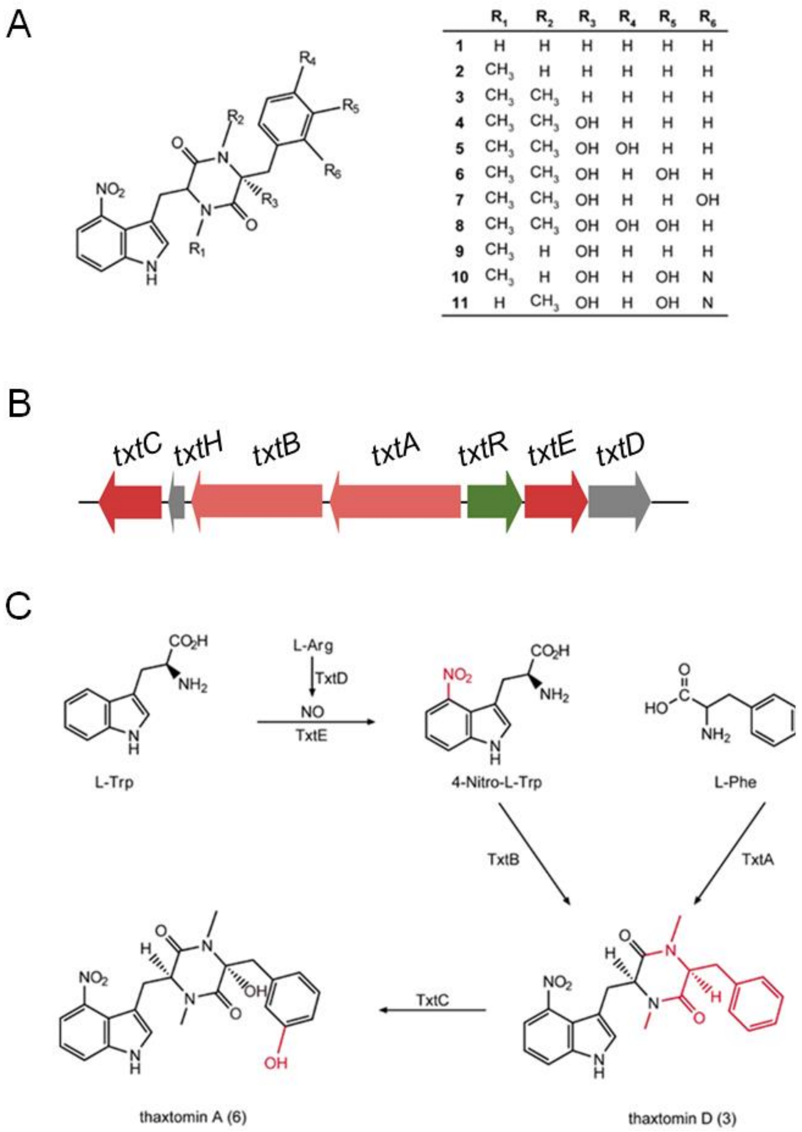

Figure S1. Thaxtomins and the biosynthetic pathway of thaxtomin A. **(A)** Chemical structures of thaxtomins. **(B)** Genetic organization of the thaxtomin A gene cluster from *S. acidsabies* ATCC 49003. **(C)** The biosynthetic pathway of thaxtomin A.

Table S1. PCR primers used in this study.

Primers	Sequence (5′---3′)
	sgRNA template primers
guide RNA-F	gttttagagctagaaatagcaagttaaaataaggctagtc
guide RNA-R	aaaagcaccgactcggtgccactttttcaagttgataacggactagccttattttaact
sgRNA-thax-up	taatacgactcactataggtacgagaaccgtaaggagccgttttagagctagaaatagcaa
sgRNA-thax-down	taatacgactcactatagggtccggccgactccaccgtagttttagagctagaaatagcaa

sgRNA-s1f	taatacgactcactataggggaattcctgccgcgatcccgttttagagctagaaatagcaa
sgRNA-s2f	taatacgactcactataggatcggcttgagcgcgcccacgttttagagctagaaatagcaa
sgRNA-s3f	taatacgactcactatagggacgccgtcacgacgccgcagttttagagctagaaatagcaa
sgRNA-s4f	taatacgactcactataggggaacgaaaactcacgttaagttttagagctagaaatagcaa
	vector amplification primers
thax-vec-F	gcggaccggttcgagtccctggcccgtactagatccttttggttcatgtgcagct
Thax-vec-R	ccgctgccgggcttgctcggagcccggcggtgaagatcctttttgataatctcat
	yeast element
URA3-F<br>URA3-R	tctacggggtctgacgctcagtggaacgaaaactcacgtgcaccacgcttttcaattca<br>aagatcctttttgataatctcatgaccaaaatcccttatggccgcatcttctcaaatatgct
	promoter cassettes
SP43	PH1F：cttccttggggtagtcgaaaagggatgcggagccggggtggccacgactttacacca-<br>tagcgcttgtccgtgtcaa
	PH1R: aatgtgaacacggatctttagacagaacgctgttcacattcgaaccgtctctgctttgacac-<br>ggacaagcgctat
SP42	PH2F：aatgtgaacagcgttctgtctaaagatccgtgttcacattcgaaccgtctctgcttt-<br>gacaacatgctgtgcggt
	PH2R： acaggtatccgttcctctctgtcgtcgagccgcccgtgttcacccgactttacaacaccg-<br>cacagcatgttgtcaa
SP30	PH3F：tcgaccaggcggtatgacgcgagacgccgtcacgacgcctgttcacattcgaac-<br>cgtctctgctttgacatcgtgtggcg
	PH3R： gtgtcgtttcctttccaagaccgcaggcctgggccgtgctggccacgactttacacccaa-<br>gcgccacacgatgtcaaagca
SP24	PM1F： cttccttggggtagtcgaaaagggatgcggagccggggtgttcacattcgaac-<br>cgtctctgctttgacaaccaag
	PM1R： aatgtgaacacggatctttagacagaacgctggccacgactttacattagatgtgcctt-<br>ggttgtcaaagcagaga
SP23	PM2F: aatgtgaacagcgttctgtctaaagatccggttcacattcgaaccgtctctgcttt-<br>gacaacatgctgtgcggtg
	PM2R: aggtatccgttcctctctgtcgtcgagccgcccgtgttagcaggactttacaacaccg-<br>cacagcatgttgtca
SP22	PM3F: tcgaccaggcggtatgacgcgagacgccgtcacgacgcctgttcacattcgaac-<br>cgtctctgctttgacaagtcgcaaaa
	PM3R: gtgtcgtttcctttccaagaccgcaggcctgggccgtgctggccacgactttacaggtt-<br>gttttgcgacttgtcaaagca
SP11	PL1F：cttccttggggtagtcgaaaagggatgcggagccggggtgttcacattcgaac-<br>cgtctctgctttgacaatggct
	PL1R： aatgtgaacacggatctttagacagaacgctggccacgactttacaccccaattcaa-<br>gccattgtcaaagcagaga
SP12	PL2F： aatgtgaacagcgttctgtctaaagatccgtgttcacattcgaaccgtctctgcttt-<br>gacaggtccatacacgcg
	PL2R： aggtatccgttcctctctgtcgtcgagccgcccgtgtggccacgactttacaagcgcgtg-<br>tatggacctgtcaa
SP10	PL3F：tcgaccaggcggtatgacgcgagacgccgtcacgacgcctgttcacattcgaac-<br>cgtctctgctttgacatgttcttacg
	PL3R: gtgtcgtttcctttccaagaccgcaggcctgggccgtgctggccacgactttacatgtgac-<br>cgtaagaacatgtcaaagca
	PCR identify
PF1	tgcacgacattgcactccac
PR1	gagctctcacgcacgctgac

PF2	acccggttcaggcaagacat
PR2	ggtctgctatgtggtgctatctg
PF3	caaggaagcccctgtgaccgt
PR3	gatcgtattcacgcgcgggatg
PF4	tgagcaaggccatcgacacgt
PR4	cgtggcggccgtactgaaat
txtEA-F	cgatggacgggtcggcgagc
txtEA-R	gcgagcgctccctccgggag
txtC-F	ccgggtcaaggcggcctgtg
txtC-R	gcgagttcgccgggtcgacc

Table S2. Sequences of promoters used in this study.

**Name**	**Sequence (5′---3′)**
SP43	tgttcacattcgaaccgtctctgctTTGACAcggacaagcgctatggtg-TAAAGTcgtggccA
SP42	tgttcacattcgaaccgtctctgctTTGACAacatgctgtgcggtgtt-gTAAAGTcgggtgaA
SP30	tgttcacattcgaaccgtctctgctTTGACAtcgtgtggcgcttgggtg-TAAAGTcgtggccA
SP24	tgttcacattcgaaccgtctctgctTTGACAac-caaggcacatctaatgTAAAGTcgtggccA
SP23	tgttcacattcgaaccgtctctgctTTGACAacatgctgtgcggtgtt-gTAAAGTcctgctaA
SP22	tgttcacattcgaaccgtctctgctTTGACAagtcgcaaaacaacctg-TAAAGTcgtggccA
SP11	tgttcacattcgaaccgtctctgctTTGACAatggcttgaattggggtg-TAAAGTcgtggccA
SP12	tgttcacattcgaaccgtctctgctTTGACAggtccatacacgcgctt-gTAAAGTcgtggccA
SP10	tgttcacattcgaaccgtctctgctTTGACAtgttcttac-ggtcacatgTAAAGTcgtggccA
